# Interfacial Tissue Regeneration with Bone

**DOI:** 10.1007/s11914-024-00859-1

**Published:** 2024-02-15

**Authors:** Stephanie S. Steltzer, Adam C. Abraham, Megan L. Killian

**Affiliations:** 1 Department of Orthopaedic Surgery, University of Michigan Medical School, Ann Arbor, MI, USA; 2 Department of Molecular & Integrative Physiology, University of Michigan Medical School, Ann Arbor, MI, USA

**Keywords:** Osteochondral interface, Enthesis, Extracellular matrix, Mechanical loading, Cellular microenvironment

## Abstract

**Purpose of Review:**

Interfacial tissue exists throughout the body at cartilage-to-bone (osteochondral interface) and tendon-to-bone (enthesis) interfaces. Healing of interfacial tissues is a current challenge in regenerative approaches because the interface plays a critical role in stabilizing and distributing the mechanical stress between soft tissues (e.g., cartilage and tendon) and bone. The purpose of this review is to identify new directions in the field of interfacial tissue development and physiology that can guide future regenerative strategies for improving post-injury healing.

**Recent Findings:**

Cues from interfacial tissue development may guide regeneration including biological cues such as cell phenotype and growth factor signaling; structural cues such as extracellular matrix (ECM) deposition, ECM, and cell alignment; and mechanical cues such as compression, tension, shear, and the stiffness of the cellular microenvironment.

**Summary:**

In this review, we explore new discoveries in the field of interfacial biology related to ECM remodeling, cellular metabolism, and fate. Based on emergent findings across multiple disciplines, we lay out a framework for future innovations in the design of engineered strategies for interface regeneration. Many of the key mechanisms essential for interfacial tissue development and adaptation have high potential for improving outcomes in the clinic.

## Introduction

Musculoskeletal disorders such as tendinopathy and osteoarthritis are some of the most prevalent nonfatal diseases. For example, in 2019, over 500 million people are afflicted with osteoarthritis worldwide [1]. Connective tissues like tendons, ligaments, and cartilage rely on integration into bone for their form and function. Despite the significant burden associated with connective tissues, our ability to repair these tissues is limited by our ability to attach or repair them with bone. This is, in part, because of the major discrepancy in mechanical properties between bone and soft connective tissues. Additionally, repair strategies have primarily focused on individual tissues, overlooking the interface and its transitional and heterogeneous structure. Musculoskeletal interfaces (e.g., osteochondral interface and tendon/ligament-bone enthesis) are transitional tissues that play an essential role in dissipating localized stresses and deformations that accumulate at sites where connective tissues meet bone. A transitional gradient from bone to connective tissue, which forms in response to applied mechanical loads during growth, is not recreated following injury and is challenging to develop in engineered constructs. A major goal of the field in interfacial biology and mechanics is to understand how these musculoskeletal interfaces can be functionally repaired and regenerated to regain their mechanical function and biological homeostasis with diminished pain postinjury. Yet how these interfaces can regenerate is poorly understood, in part because of our limited understanding of how these tissues develop and heal following injury.

In this review, we highlight some of the current progress made in understanding the development of interfacial tissue and their adaptation during growth and following injury and share new innovations and approaches for interfacial tissue regeneration. We focus primarily on emerging challenges for studying the osteochondral interface and the enthesis revolving around the cellular (e.g., differentiation and metabolism) and extracellular matrix (ECM) processes (e.g., which contribute to its mechanical environment). We also discuss potential tools to advance knowledge in regenerative approaches for interfacial tissue regeneration, primarily through interfacial tissue development.

## Cues from Interface Development to Guide Regenerative Approaches

A major challenge of interface regeneration is re-establishing the cellular and structural heterogeneity of healthy interfaces. This challenge arises from complexities in structure and function, as each interface has unique cellular and biomechanical demands [2]. Resident cells of tissue interfaces contribute to the establishment, remodeling, and maintenance of the interface, such as the ECM gradient that defines an osteochondral or tendon-bone interface. Interfacial tissues lack the innate ability to regenerate, in part because of dense ECM, low cellular density, and poor vascular supply. Guided regeneration will require novel engineered approaches to promote integration of one tissue with the other. For example, a cellular gradient exists at the tendon-bone enthesis (e.g., tenocytes, fibrochondrocytes, and osteocytes), and these cells remodel and deposit their local ECM (e.g., collagen types I, II, and X, and proteoglycans), which contributes to their local mechanical environment (e.g., stiffness). Platform-based engineered tissues have emerged to evaluate these dynamics by exploiting compressive boundary conditions in vitro [3•, 4]. Cells within interfacial tissues locally establish and remodel their surrounding environment by degrading or depositing new matrix. In the developing tendon-bone enthesis, cellular density is highest during its early growth phase, before which an organized, gradient ECM is established [5]. As the cells deposit ECM, a functional gradient forms at the tendon-to-bone insertion [6], and at the same time, cellular density decreases [5]. Interfacial tissue healing during enthesis development has recently shown that the tendon-bone enthesis also has innate regenerative properties which are not modeled in adult animals [7–9].

Post-injury in the neonatal enthesis, the injured tissue is hypocellular and avascular [9]. In tendon, Grinstein et al. demonstrated that tendon cells shift from rates of high to low proliferation during postnatal growth in mice, and expression of genes associated with tendon transcription factors and ECM decreases with age [10]. Additionally, neonatal tendon cells express markers of mesenchymal stem cells (MSC) but differ from bone-marrow MSCs as neonatal tendon cells demonstrate reduced differentiation potential toward chondrogenesis and osteogenesis compared to bone-marrow MSCs [11, 12]. Results from these studies reveal progenitor cells that have undergone differentiation toward tendon cells are terminal, introducing a potential challenge for interfacial tissue regeneration, as terminally differentiated cells post-injury may not have the high regeneration potential of progenitor cells typically involved during neonatal development. Thus, it is essential to understand the cellular mechanisms involved in interfacial tissue development that can be used to drive regeneration using native cell types.

Tools such as transgenic animals for lineage tracing, flow cytometry and cell sorting, and RNA sequencing have been foundational for understanding how the resident cell population establishes the interface. For example, it is known that cells rely on the expression of the transcription factor *Scleraxis* (*Scx*^+^) for enthesis development [13, 14]. Additionally, *Scx*^+^ cells that co-express *SRY-box transcription factor 9* (*Sox9*^+^) are bi-fated and generate both *Scx*^+^ tendon fibroblasts and *Sox9*^+^ chondrocytes critical for enthesis formation (Fig. 2) [15, 16••]. Recent work by Best and Loiselle showed that *Scx* lineage cells are essential for generating an organized and bridging tissue following tendon injury, and ablation of *Scx*-lineage cells may improve tendon healing [17]. However, *Scx* + cells are required for adult tendon homeostasis [17–20], and expression of *Scx* is required to recruit mesenchymal progenitors during embryonic tendon elongation and regeneration in both mice and zebrafish [21–23]. *Scx*^+^ cells are also responsible for healing neonatal tendons [24, 25].

The hedgehog (Hh) signaling pathway is critical for formation and maintenance of tissue interfaces [7, 26–29]. For example, Felsenthal et al. found that *Sox9*^+^ lineage cells are replaced with cells expressing *Glioma-associated oncogene homolog 1* (*Gli1*^+^), a hedgehog-responsive transcription factor [30, 31]. These initial *Sox9*^+^ progenitor cells are necessary to establish the fibrocartilaginous template before cells are removed and replaced (during attachment migration) or further differentiate into *Gli1* + cells [30] [32]. *GLI family zinc finger 3* (*Gli3*) is also an essential regulator of patterning of attachment-site progenitors [33]. These findings during enthesis development support the balance *Scx*, *Sox9*, and hedgehog pathways for establishing the cell gradient necessary for enthesis function. Another emergent signaling pathway involved in post-natal interface development is fibroblast growth factor (FGF) signaling, in part because it plays a key role in mineralization at the tendon-to-bone enthesis and regulates cell fate in fibrocartilage tissues [5, 34–37]. Mechanobiological processes associated with cilia have recently been deemed critical for enthesis formation and healing and are mediated via Hh signaling and mechanical loading [28, 38–40]. The regulation of ECM deposition by mechanical loading and cellular pathways like Hh and FGF signaling suggests these are key targets and tools for regenerative approaches for the interface.

## Contributions of the Local ECM on Interfacial Development and Healing

**Fig. 1:**
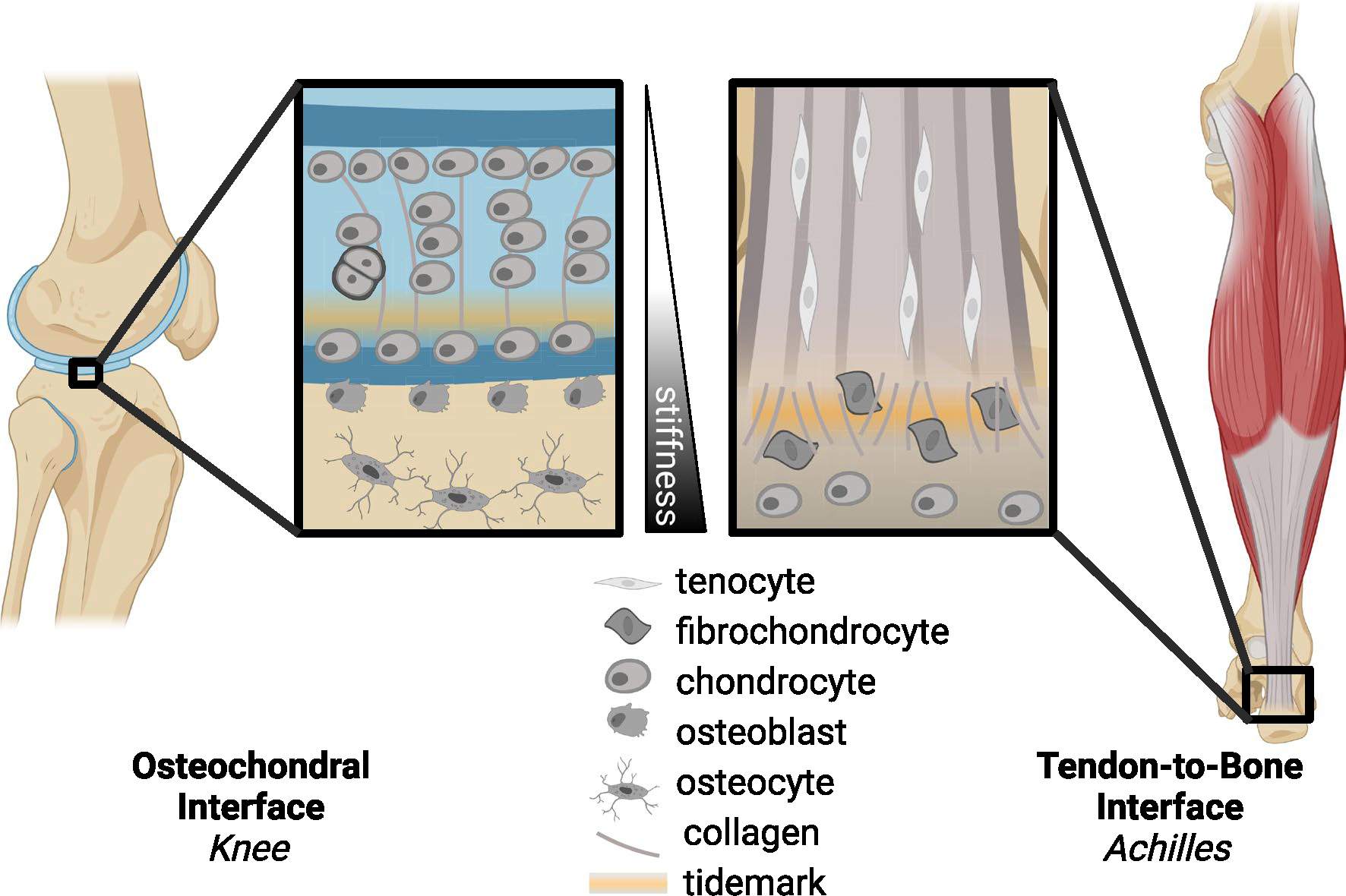
Schematics of the osteochondral interface (e.g., within the knee) and the tendon-to-bone interface (e.g., Achilles enthesis), which highlight the stiffness gradients (from bone to tendon), tidemark between mineralized and unmineralized fibrocartilage, variations in collagen alignment and fibril size, and cell type distribution inclusive of tendon fibroblast, fibrochondrocyte, and chondrocytes/osteoblasts

**Fig. 2:**
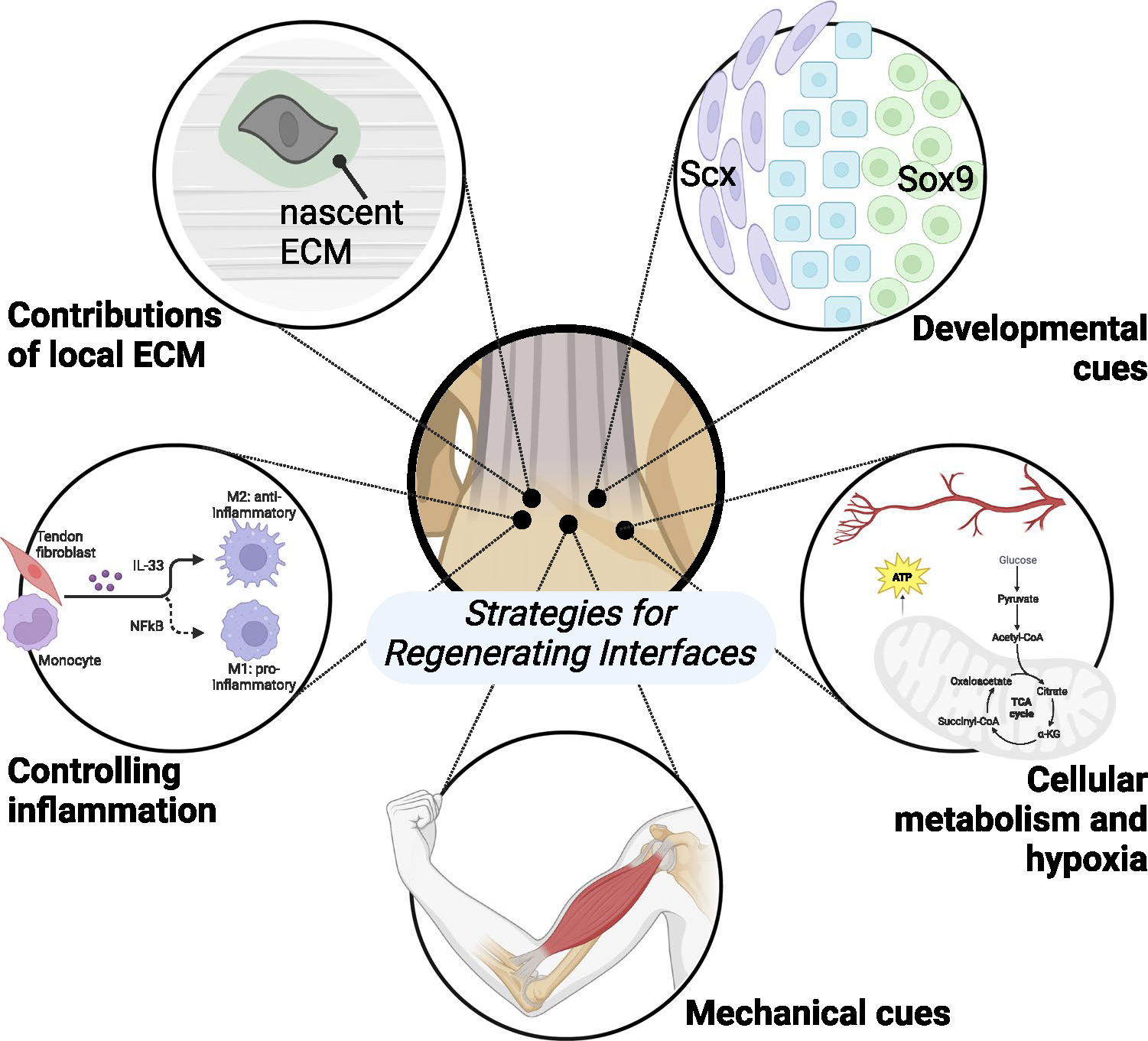
Strategies for regenerating interfacial tissues like the osteochondral interface and tendon-bone enthesis include: leveraging developmental cues to promote resident and progenitor cell remodeling of the interfacial tissues (e.g., Sox9+/Scx+bi-fated cells, shown in blue, residing between Scx+cells in purple and Sox9+cells in green); identifying factors that influence formation of nascent ECM in native tissues during remodeling and repair; promoting a regenerative, rather than destructive, inflammatory response; controlling the mechanical environment by increasing or decreasing applied loads (e.g., from skeletal muscle); and understanding and controlling interfacial cell metabolism and physiological response and sensitivity to their environment, such as hypoxia

Cell-ECM interactions regulate mechanotransduction, particularly within key transitional tissues such as the osteochondral interface or the enthesis (Fig. 2) [41]. Mechanical and chemical cues are transduced from ECM to the cells to regulate production of nascent ECM. Remodeling of the ECM by matrix metalloproteases (MMPs) and tissue inhibitor of metalloprotease (TIMPs) and deposition of nascent ECM are critical to maintaining tissue homeostasis throughout changes in mechanical loading such as tension, compression, shear, and hydrostatic strains [42]. Within the enthesis, there is a complex ECM gradient from the tendon into the bone including a transition of primarily collagen types I and II, with collagen types III, V, VI, X, and XI contributing to the complex collagen and mineralized gradient [43–45]. Similarly, the osteochondral interface is also characterized by its unique cellular and ECM gradient [46, 47]. The mineralized and unmineralized regions of these interfaces are separated by a distinct tidemark, and the collagen organization and fibril size change, becoming smaller during the transition from soft to hard matrix (Fig. 1). Our ability to visualize and quantify specific spatial qualities of interfaces has been improved with refined techniques in fractionation, mass spectrometry, and proteomics in other interfacial tissues, like the myotendinous junction [48•]. The inability to remodel adult extracellular and pericellular matrix is a major obstacle to overcome in the context of interfacial tissue healing. The remodeling of ECM in mature interfaces is limited, in part, by increased collagen cross-linking due to advanced glycation end products (AGEs) or lysyl oxidase (LOX), increased ECM-to-cell ratio, and production, or lack thereof, of pericellular matrix [49–53]. Interfacial tissue ECM is also rich in proteoglycans, providing this tissue with osmotic properties to reduce compressive stress. Using hyperelastic characterization of strain-stiffening in cartilage, McCreery et al. found proteoglycans drive the strain-stiffening response in hyaline cartilage [54]. These data support that ECM components are key to mechanosensing and mechanical function of interfacial tissues.

ECM composition may also play role in stimulating inflammation [55]. While inflammation is a key response to injury, chronic inflammation and accumulated fibrotic ECM can hinder the ability of interfacial tissue to regenerate. Clues from neonatal healing may provide insights of the regenerative potential of remodeling or maintenance of the native ECM by tissue resident cells, as a recent study by Vinestock et al. has shown that neonatal enthesis injury leads to generation of an acellular and low-inflammatory scar driven primarily by resident cells [9]. The intricate crosstalk between cells and their nascent ECM may influence the inflammatory response involved in tissue remodeling.

### Controlling Inflammation to Influence Interfacial Tissue Healing

The inflammatory response contains a myriad of different cell types and functions making it challenging to decouple the beneficial or harmful mechanisms post-injury on interfacial tissues (Fig. 2) [56]. Inflammation may cause disruptions in homeostasis causing modulations in the native ECM architecture and mechanics that are challenging to reverse [42]. Regulatory T cells (Tregs) impact resident cells, and neonates have elevated levels of Tregs to help prevent autoimmune response [24, 57]. Tregs maintain the environment necessary for the switch from pro-inflammatory to anti-inflammatory macrophage phenotypes necessary to regulate tissue regeneration [55, 58]. Howell et al. found macrophages to be critical for neonatal tendon regeneration [24, 59]. Biomaterials can be designed to employ immunomodulatory effects to reduce the localized overactive immune response.

Cytokines, particularly IL-33, have been studied in the context of tendon and enthesis injury as well [60]. IL-33 expression is elevated in the human torn tendon and in early tendinopathy [60, 61]. Additional studies interrogating the inflammatory response post-injury or during disease progression (e.g., enthesitis) have revealed non-autonomous functions driving healing [18, 62], such as the protein complex known as nuclear factor kappa-light chain-enhancer of activated B cells (NF-kB) [63–65].

## Leveraging Mechanical Cues to Understand Interfacial Tissue Development

The primary function of tissue interfaces is to transmit and dissipate forces between tissues with dissimilar mechanical properties (Fig. 2). In the absence of mechanical loading during periods of growth, these tissues do not form their hallmark gradient cellular and ECM morphology and are also remarkably weaker and less mechanically resilient [6, 13, 66]. Mechanical loading (e.g., from skeletal muscle contractions) can regulate cell-scale ciliary Hh signaling [26, 28, 29], interfacial matrix organization [6, 13, 67, 68], and accrual of mineral [6, 66]. Both the organization and deposition of mineral drive the mechanical toughness at the interface [66]. Furthermore, mechanical loading stimulates primary cilia assembly, which is required for Hh signaling [28]. These findings can be used in regenerative medicine approaches by mimicking loading using Hh activation via biomaterials or small molecules.

At the microscale, the ability of cells to respond to local substrate stiffness has been investigated for decades, yet our understanding of how cells interact within gradient materials is relatively new [69, 70]. Key studies have demonstrated the importance of loading in interface disorders such as rotator cuff disease and ligament repair [71–73]. At the cellular level, changes in stiffness elicit transcriptional changes which can lead to changes in cell fate [74–78]. Furthermore, tendon stromal compartments respond to mechanical unloading dependent on the vascular niche as well as reactive oxidative species (ROS) which can proteolytically break down functional collagen backbones [79]. Mechanical force has also been shown improve rotator cuff tendon-bone healing by activating the IL-4/JAK/STAT signaling pathway through mediation of macrophage polarization, indicating a feedback system involving both mechanosensing and the immune system [58, 80]. Therefore, in order to regenerate this tissue, strategies must consider mechanosensing of resident cells at tissue interfaces [81].

## How Are Cellular Metabolism and Hypoxia Involved in Interface Healing?

Metabolism is a key driver of changes within the cell as it is typically the first approach for cells to adapt to changes in their environment, such as changes in oxygen availability and vasculature. Yet the metabolic profile of cells at tissue interfaces is poorly understood and a prime target for future studies. In cartilage, suppression of mitochondrial respiration is a key driver for chondrocyte survival under hypoxic conditions [82, 83]. In tendon, disorders such as tendinosis have been associated with changes in oxygen tension-dependent modulation of Rac1 activity [84]. Hypoxia inducible factor 1a (HIF-1α) is an oxygen-dependent transcription factor that regulates gene expression of genes affiliated with metabolism, angiogenesis, and matrix maturation. HIF-1α has recently gained more traction in the study of hypoxic, ECM-rich tissues such as the cartilage and tissue interfaces, and has potential to act as a therapeutic target for treating osteoarthritis [85]. HIF-1α metabolically regulates collagen synthesis and modification in chondrocytes [86]; thus, it may contribute to the establishment of the ECM gradient in the fibrochondrogenic enthesis (Fig. 2). In vitro, the deposition of osteochondrogenic matrix is mediated by HIF-1α in hypoxia [87]. However, prolonged HIF-1α signaling in chondrocytes via HIF prolyl hydroxylase 2 (PHD2) deactivation restricts cellular bioenergetics and biosynthesis, leading to skeletal dysplasia [86]. In addition to the metabolic response to changes in oxygen availability, increased matrix production is correlated with decreased mitochondrial gene expression as well as a lack of inflammatory signature [73]. With the close ties between oxygen availability and vascularity, studies focused on the effects of vascularity on cell fate within interfacial tissue are of particular interest. For instance, vascularity and lipid availability regulate skeletal progenitor cell fate while *Sox9* suppresses fatty oxidation in chondrocytes [88]. More studies investigating vasculature in interfacial tissue are required to better inform regenerative approaches.

## Impact in Discovery and Clinical Translation

Cues from interfacial tissue development and healing have the potential to inform how we treat and repair interfaces. One strategy for regeneration that has shown promise is biomaterial scaffolds; however, these strategies have been used for decades with limited translation to the clinic, and few of these are focused on complex tissue interfaces. To stimulate interfacial tissue regeneration, biomaterial design could promote a microenvironment like that of the developing interfacial tissue. To advance biomaterial design, continued study of interface development and healing is necessary, in addition to assessment of cell behavior in gradient-structured materials. To this end, biofabrication of interfaces highlights major advances in the field of tissue engineering, including development bi-zonal patterning of engineered constructs [89, 90] and preclinical translation of composite biomaterials for craniofacial osteochondral repair [91] and tendon-bone enthesis repair [92–94] have shown promise.

Biomaterials have proven to be a more popular method for interfacial tissue repair as they offer a more controllable system without the complexity of cell implantation (i.e., cells are challenging to scale-up production reproducibly for transplant) [95, 96]. There are several approaches to biomaterial design for interfacial tissue regeneration including synthetic vs. natural constructs, injectable vs. non-injectable materials, 3D-printed vs. electrospun, degradable vs. non-degradable, and multi-phasic vs. uni-phasic [97–104, 105••]. Currently, substantial progress has been made in osteochondral interfacial regeneration with bone using biomaterials, particularly in craniofacial bone defects [91, 97, 106–109]. These provide further motivation to investigate the use of biomaterials to heal the osteochondral interface or enthesis. As interfacial tissues with bone are graded, collagen-rich tissues, collagen scaffolds have been of particular interest to guide native cellular migration and spatial differentiation at the defect site. However, synthetic materials may provide a better scaffold for cells to interact with their microenvironment and generate the collagen or ECM required for regenerating the gradient. Overall, non-cellularized or acellularized multiphasic scaffolds generated to treat injured interfaces show promise in vitro, but require validation in vivo [110].

Interfacial tissue proves to be challenging to regenerate; however, increased studies in ECM remodeling, the immune system, mechanical loading, metabolism, hypoxia, and angiogenesis in development and healing will provide new insights into regenerative approaches. Cell functions within interfacial tissues contribute to the unique mechanical properties. ECM components derived from cells are key to mechanosensing and mechanical function of interfacial tissues. Furthermore, studies support that the tissue regeneration and healing can be regulated by mechanical force, the immune system, and metabolism as well. Knowing this, we can tune regenerative approaches using biomaterials or pharmaceuticals to promote interfacial tissue regeneration post-injury as we continue to study interfacial tissue development and healing.
